# Supplementary material for: The Brazilian System for Monitoring Workers and General Population Exposed to Asbestos: Development, Challenges, and Opportunities for Workers’ Health Surveillance
Source: Int J Environ Res Public Health. 2023 Feb 28;20(5):4295. doi: 10.3390/ijerph20054295 (PMC10001436; doi:10.3390/ijerph20054295)
Supplement: Supplementary file 1 [file ijerph-20-04295-s001.zip › ijerph-2194763-supplementary.pdf]

DATAMIANTO<br>Brazilian system for monitoring workers and general population exposed to asbestos	RECORD<br>Nº_____________

REGISTRATION SERVICE

Registration date: ___/___/____ Responsible for filling in: ....

Center control: ....

OCCUPATIONAL HEALTH SURVEILLANCE ACTION

Action date: ___/___/____ ☐ Action taken by the Company ☐ Action taken by Occupational Health Surveillance service

Company's name: ....

The company sent mail acknowledgment of receipt (AR): ☐ Yes ☐ No ☐ Not applicable

AR registration date: ___/___/___ AR registration nº: ....

AR return date: ___/___/___

Reason for returning the AR: ☐ Absent ☐ Unknown ☐ Insufficient address ☐ Deceased ☐ Outside the urban perimeter ☐ Moved ☐ Not wanted ☐ Number does not exist ☐ Declined

The company carried out the recommended tests: ☐ Yes ☐ No ☐ Not applicable

☐ Clinical assessment ☐ Spirometry ☐ Chest X-ray

The company summoned the worker: ☐ Yes ☐ No ☐ Not applicable

The worker was informed about the next return date (there is a way to prove): ☐ Yes ☐ No ☐ Not applicable

The company actively search for new cases: ☐ Yes ☐ No ☐ Not applicable

Did the company issue an Occupational Health Certificate (ASO)?

In admission: ☐ Yes ☐ No – If yes, Date:___/___/____ - Acknowledged asbestos exposure: ☐ Yes ☐ No

Periodical: ☐ Yes ☐ No – If yes, Date:___/___/____ - Acknowledged asbestos exposure: ☐ Yes ☐ No

Change of function: ☐ Yes ☐ No – If yes, Date:___/___/____ - Acknowledged asbestos exposure: ☐ Yes ☐ No

Return to work: ☐ Yes ☐ No – If yes, Date:___/___/____ - Acknowledged asbestos exposure: ☐ Yes ☐ No

Dismissal: ☐ Yes ☐ No – If yes, Date:___/___/____ - Acknowledged asbestos exposure: ☐ Yes ☐ No

Other information:

Fill in if the health surveillance action **was performed by the company:**

☐ The company considers that it has **fully** complied with the legislation (Annex 12 of NR 15)

☐ The company considers that it has **partially** complied with the legislation (Annex 12 of NR 15)

☐ The company considers that it has **not** complied with the legislation (Annex 12 of NR 15)

Fill in if the health surveillance action **was performed by Occupational Health Surveillance service:**

☐ The company **fully** complied with the legislation (annex 12 of NR 15)

☐ The company **partially** complied with the legislation (annex 12 of NR 15)

☐ The company **not** complied with the legislation (annex 12 of NR 15)

ASBESTOS EXPOSURE

Name of the exposed individual: ....

Date of birth: ___/___/____ **Age**: **Weight (Kg)**: **Sex:** ☐ Male ☐ Female ☐ Other ☐ Unknown

Pregnant woman: ☐ 1st trimester ☐ 2nd trimester ☐ 3rd trimester ☐ Unknown trimester ☐ Unknown ☐ No ☐ Not applicable

Race: ☐ White ☐ Black ☐ Yellow ☐ Brown ☐ Indigenous ☐ Unknown

Education level: ☐ Illiterate ☐ Elementary school complete ☐ Elementary school incomplete ☐ Secondary school complete ☐ Secondary school incomplete ☐ Higher education complete ☐ Higher education Incomplete ☐ Not applicable ☐ Unknown

Residential address: Street: .... nº .... Complement: .... Neighborhood: ....

ZIP Code: .... Municipality: .... State: .... Country: ....

CONTACTS – Type of contact: (Email/Fax/Other/Cell Phone/Bussiness Phone/Landline Phone): ....

Family contacts

Contact name: .... **Type of relationship:** ....

Date of birth: .... **Contact**: ....

Complementary data

Mother´s name: ....

Identification number of exposed (CPF): .... **National health registry (CNS) of exposed**: ....
Worker Identification Number (PIS/PASEP): .... **Identification number (RG):** ....
Registry number: .... **Health insurance:** .... **Work and Social Security Card (CTPS):** ....

PATIENT'S HISTORY

Date of action: ___/___/____ **Time**: .... **Health Service/Center:** .... **Responsible:** ....

AGENT (TYPE OF ASBESTOS)

Date of action: ___/___/____ **Type of Asbestos:** ☐ Actinolite ☐ Amosite ☐ Indeterminate Amphibole ☐ Antophyllite ☐ Chrysotile ☐ Crocidolite ☐ Tremolite ☐ Indeterminate

EXPOSURE INFORMATION

Circumstance: ☐ Occupational ☐ Para-occupational ☐ Residential ☐ Environmental ☐ Indeterminate
☐ Other occupational exposure to dust (non-Asbestos)

- **If occupational exposure:**

Date of action: ___/___/____ Dismissal date/end of contract: ___/___/____
Start date (month and year): ____/____ Finish date (month and year): ____/_____
☐ Individual remains exposed
Type of work bond: ☐ Formal ☐ Informal
<u>Formal bond:</u>
Company's name: ....
Function: .... Complementary Function (CBO): ....
<u>Informal bond:</u>
Economic activity (CNAE): ....
Occupation: .... Complementary Occupation (according to CBO): ....

- **If para-occupational exposure:**

Date of action: ___/___/____ Dismissal date/end of contract: ___/___/____
Start date (month and year): ____/____ Finish date (month and year): ____/_____
☐ Individual remains exposed
Type of work bond: ☐ Formal ☐ Informal
<u>Formal bond:</u>
Company's name: ....
Function: .... Complementary Function (CBO): ....
<u>Informal bond:</u>
Economic activity (CNAE): ....
Occupation: .... Complementary Occupation (according to CBO): ....

- **If residential exposure:**

Date of action: ___/___/____ Relationship to the exposed individual: ....
Start date (month and year): ____/____ Finish date (month and year): ____/_____
☐ Individual remains exposed
Type of work bond: ☐ Formal ☐ Informal
Registration number of exposed individual: ....
Company's name: ....

- **If environmental exposure:**

Date of action: ___/___/____
Start date (month and year): ____/____ Finish date (month and year): ____/_____
☐ Individual remains exposed
Proximity to the company: .... (meters/kilometers)
Company's name: ....

- **If indeterminate exposure:**

Date of action: ___/___/____ Notes: ....

- **Other Occupational Dust Exposure Non-Asbestos Circumstance**

Date of action: ___/___/____ Dismissal date/end of contract: ___/___/____
Start date (month and year): ____/____ Finish date (month and year): ____/_____
☐ Individual remains exposed

Type of work bond: ☐ Formal ☐ Informal

Formal bond:

Company's name: ....

Function: .... Complementary Function (CBO): ....

Informal bond:

Economic activity (CNAE): ....

Occupation: .... Complementary Occupation (according to CBO): ....

Type of dust: (Charcoal/Hard Metals/Other Dusts/Other Metals/Organic Dusts/Free Silica/Silicates)

SMOKING HABITS

Date of action: ___/___/____

Ever smoked: ☐ Yes ☐ No ☐ Unknown

If yes, currently smoking: ☐ Yes ☐ No ☐ Unknown - Cigarettes per day: ....

Age that started smoking: .... How many years ago did you stop smoking: .... PACK/YEARS: ....

CLINICAL MANIFESTATIONS

RESPIRATORY SYMPTOMS

Date of action: ___/___/____

- **COUGH** - Do you usually cough when waking up: ☐ Yes ☐ No ☐ Unknown

If yes, do you cough at least 3 months a year upon awakening: ☐ Yes ☐ No ☐ Unknown

Do you usually cough during the day or night: ☐ Yes ☐ No ☐ Unknown

- **PHLEGM** - Do you usually spit upon waking up: ☐ Yes ☐ No ☐ Unknown

If yes, do you spit at least 3 months a year after waking up: ☐ Yes ☐ No ☐ Unknown

Do you usually spit during the day or night: ☐ Yes ☐ No ☐ Unknown

How many years have you had morning cough and phlegm: .... (years)

- **DYSPNEA** – Feel shortness of breath (dyspnea): ☐ Yes ☐ No ☐ Unknown - If yes, for how long: .... (days/months/years)

Do you feel dyspnea when exercising: ☐ Yes ☐ No ☐ Unknown

Do you feel dyspnea when walking fast: ☐ Yes ☐ No ☐ Unknown

Can you keep up with people of similar age, walking normally on the flat ground: ☐ Yes ☐ No ☐ Unknown

Do you have to stop to rest/breathe when walking on flat ground at a normal pace, after a few minutes or about 100 meters: ☐ Yes ☐ No ☐ Unknown

Do you feel shortness of breath that you do not leave house anymore, or when you are getting dressed: ☐ Yes ☐ No ☐ Unknown

- **WHEEZING** – Do you have wheezing: ☐ Yes ☐ No ☐ Unknown

CONSTITUTIONAL SYMPTOMS

Date of action: ___/___/____

WEIGHT LOSS: ☐ Yes ☐ No ☐ Unknown – If yes, how many kilos and in how long: .... Kg ....(days/months/years)

FEVER: ☐ Yes ☐ No ☐ Unknown

BACKGROUND HISTORY

Date of action: ___/___/____

• **DISEASES**

Heart diseases: ☐ Yes ☐ No ☐ Unknown

Neoplasms (cancers/tumors): ☐ Yes ☐ No ☐ Unknown – If yes, which ones: ....

Lung diseases: ☐ Tuberculosis ☐ Bronchitis-Asthma ☐ Pneumonia ☐ Pleural effusion ☐ Chest trauma

Other lung diseases: ☐ Yes ☐ No ☐ Unknown – If yes, which one: ....

Have you ever had any chest surgery: ☐ Yes ☐ No ☐ Unknown

Have you ever attended a doctor appointment for a lung/respiratory problem: ☐ Yes ☐ No ☐ Unknown

If yes, which symptoms/diseases: .... **For how long:** .... (days/months/years)

Other important diseases: ....

IMAGE EXAMS

Have you ever had a chest X-ray or X-ray: ☐ Yes ☐ No ☐ Unknown

Have you ever had a chest tomography: ☐ Yes ☐ No ☐ Unknown

If yes, do you have these images or exams at home: ☐ Yes ☐ No ☐ Unknown

---

COMPLEMENTARY EXAMS

(Upload of PDF, AVI, WMV, MP4, JPG/JPEG, PNG, BMP, XLS/XLSX, DOC/DOCX e DCM files are accepted – max. size of 50MB per file)

Types of exams: ASBESTOS/PATHOLOGICAL ANATOMY/IMAGE/LABORATORY/OTHER/ALL Date: ___/___/____

(Note: for each 'type of exam' selected, several exams will be listed. Follow an example when selecting 'type of exam: asbestos')

Available exams (for asbestos):

☐ Asbestos – other
☐ Pleural biopsy by pleuroscopy
☐ Pleural biopsy by pleuroscopy with immunohistochemistry for MMP
☐ Pleural biopsy/thoracentesis
☐ Pleural biopsy/thoracentesis with immunohistochemistry for MMP
☐ Open lung biopsy
☐ Video-assisted thoracoscopy lung biopsy
☐ Transbronchial biopsy
☐ Transthoracic biopsy under imaging
☐ Bronchoscopy
☐ DL-CO
☐ Spirometry
☐ Mediastinoscopy/mediastinostomy
☐ PET-CT
☐ Plethysmography
☐ Chest X-ray
☐ 6-minute walking test
☐ Chest computed tomography

Include exam results:

ANNEXES

(Medical report / OIT radiological reading report 2011 / CAT / SINAN / Post-dismissal medical assessment / Clinical record / Medical assessment / Social insurance decision communication / Declaration / Worker's convocation proof / Patient's referrals / Medical prescription / Death certificate and/or Death declaration / Exams / Admission-Periodic-Dismissal assessment – ASO / Employee registration form / Other)

(Upload of PDF, AVI, WMV, MP4, JPG/JPEG, PNG, BMP, XLS/XLSX, DOC/DOCX e DCM files are accepted – max. size of 50MB per file)

PATIENT'S DIAGNOSIS

New Diagnosis

Diagnosis date: ___/___/____

Diagnosis

☐ Asbestosis ☐ Laryngeal carcinoma ☐ Ovarian carcinoma ☐ Lung carcinoma ☐ Non-asbestosis interstitial disease
☐ Asbestos-related pleural disease ☐ Chronic airflow limitation ☐ Peritoneum mesothelioma
☐ Pleural mesothelioma ☐ Others sites mesothelioma ☐ Undiagnosed

☐ Absence of asbestos-related pleuro-pulmonary disease

☐ Other diagnosis - If other diagnosis, inform ICD-10: ....

Severity rating: ☐ Mild ☐ Moderate ☐ Severe ☐ Fatal ☐ Unknown

---

WORK ACCIDENT REPORT (CAT) and NOTIFIABLE DISEASES INFORMATION (SINAN)

Date of action: ___/___/____

Add new CAT

Date of action: ___/___/____ CAT has been opened? ☐ Yes ☐ No ☐ Not applicable

Responsible for the CAT opening? ☐ Company ☐ Other institution ....

CAT number: .... CAT issue date: ____/____/_____

Date of action: ___/___/____ SINAN has been opened? ☐ Yes ☐ No ☐ Not applicable

SINAN notification number: .... Date of SINAN registry: ____/____/_____

REVALUATIONS

Routine reassessment schedule

Date: ___/___/____ Hour: _____________ ☐ Consider smoking on periodic reassessment *(exclusive for the São Paulo State, according to the State Resolution SS 70/2020).*

Referrals: ....

Description: ....

CLOSING OF PATIENT'S RECORD

Death certificate data

Date of Death: ___/___/____ **Age:** ....

Death certificate number: ....

**Death certificate – Original**	**Death certificate - rectified field(s) after Epidemiological Investigation**
	Date of completion of the investigation: ___/___/____<br>Research source: *Home visit - interview with the family / Medical Records / Information database (SUS): SIH, SIA, SIM, SINAN / SVO / IML / Multiple sources / Other sources.*
**Part I**<br>a. Immediate or terminal cause<br>b. Intermediate cause<br>c. Intermediate cause<br>d. Basic cause of death	**Part I**<br>a. Immediate or terminal cause<br>b. Intermediate cause<br>c. Intermediate cause<br>d. Basic cause of death
**Part II**<br>a. Immediate or terminal cause<br>b. Intermediate cause<br>c. Basic cause of death	**Part II**<br>a. Immediate or terminal cause<br>b. Intermediate cause<br>c. Basic cause of death

Outcomes:

Death – Relationship with asbestos:

☐ Death due to asbestos exposure

☐ Death from other causes

☐ Death under investigation

☐ Death not investigated
